# Real-Time Detection of Strawberry Ripeness Using Augmented Reality and Deep Learning

**DOI:** 10.3390/s23177639

**Published:** 2023-09-03

**Authors:** Jackey J. K. Chai, Jun-Li Xu, Carol O’Sullivan

**Affiliations:** 1 School of Computer Science and Statistics, Trinity College Dublin, D02 PN40 Dublin, Ireland; chaij@tcd.ie (J.J.K.C.);; 2 School of Biosystems and Food Engineering, University College Dublin, D04 V1W8 Dublin, Ireland

**Keywords:** strawberry, ripeness, augmented reality, deep learning, YOLOv7

## Abstract

Currently, strawberry harvesting relies heavily on human labour and subjective assessments of ripeness, resulting in inconsistent post-harvest quality. Therefore, the aim of this work is to automate this process and provide a more accurate and efficient way of assessing ripeness. We explored a unique combination of YOLOv7 object detection and augmented reality technology to detect and visualise the ripeness of strawberries. Our results showed that the proposed YOLOv7 object detection model, which employed transfer learning, fine-tuning and multi-scale training, accurately identified the level of ripeness of each strawberry with an mAP of 0.89 and an F1 score of 0.92. The tiny models have an average detection time of 18 ms per frame at a resolution of 1280 × 720 using a high-performance computer, thereby enabling real-time detection in the field. Our findings distinctly establish the superior performance of YOLOv7 when compared to other cutting-edge methodologies. We also suggest using Microsoft HoloLens 2 to overlay predicted ripeness labels onto each strawberry in the real world, providing a visual representation of the ripeness level. Despite some challenges, this work highlights the potential of augmented reality to assist farmers in harvesting support, which could have significant implications for current agricultural practices.

## 1. Introduction

The strawberry fruit is favoured by consumers because it is a good source of antioxidants and nutrients [1]. Strawberries are also widely cultivated and rank first worldwide among the production of small berries [2]. In 2021, the worldwide market for fresh strawberries held a valuation of approximately USD 24.79 billion, and it is anticipated to ascend to around USD 43.33 billion by the year 2028 [3]. Ripe strawberries are sweet and juicy, possessing considerable economic worth. Given that strawberries are categorised as non-climacteric fruits—meaning they exclusively mature while attached to the plant—it becomes imperative to harvest them during their prime ripeness to guarantee the fruit’s superior quality [4]. Despite continuous endeavours aimed at creating robotic harvesting solutions for strawberries and various other crops, the realisation of a fully functional commercial system remains elusive. As a consequence, the present process of strawberry harvesting continues to depend heavily on human labour. After the harvesting process, growers primarily evaluate the ripeness of strawberries by tallying the cultivation period, inspecting the colours of the fruits, and sometimes relying on personal taste judgment. However, this human element introduces subjectivity, resulting in an uneven ripeness among post-harvest strawberries, which significantly undermines the overall quality and uniformity of the strawberries [5]. To this end, there exists a considerable market demand for the automated identification of strawberry ripeness within the field, aiming to enhance the precision of selective harvesting processes [6]. 

Artificial intelligence has garnered growing interest across various domains and has been integrated into agricultural practices to advance the automation of production processes [7]. Over the past few years, deep learning (DL) has surfaced as a catalyst, propelling artificial intelligence to new heights, and offering optimal solutions to numerous challenges within the realm of image recognition [8]. Conventional machine learning approaches necessitate the manual curation of features that classifiers utilise for identifying patterns. While effective for addressing straightforward or clearly defined issues, these methods often falter when confronted with intricate real-world challenges, such as object detection. In contrast, deep learning is specifically engineered to transcend this constraint, employing intricate neural networks that empower computers to perceive, comprehend, and respond to intricate scenarios. In terms of ripeness detection, Miragaia et al. [9] developed a classification system to determine the ripening stage of plums based on Convolutional Neural Networks (CNN). Additional research has documented the utilisation of CNN for categorising the various ripening stages of apples [10], mulberries [11], and bananas [12]. 

Strawberries are usually difficult to detect due to the significant variability among fruits (e.g., size and colour) [13]. Detecting strawberries involves the initial step of locating a strawberry fruit within an image or video. This process of object detection proves valuable for enumerating instances of strawberries within a scene and monitoring their exact positions. While various studies have documented the use of Region-based Convolutional Neural Networks (R-CNN), these are deemed impractical for real-time applications due to the significant time required to execute object detection [14]. Conversely, the You Only Look Once (YOLO) approach [15,16] offers a substantial enhancement in detection speed as a one-stage detector. This method operates by employing the entire image as the network’s input and directly provides output pertaining to the bounding box positions and the associated class probabilities of those bounding boxes [17], thereby enabling real-time object detection. In contrast to its initial iteration, YOLOv2 introduced a novel integrated training approach that enables users to train object detectors using both detection and classification data [16]. Subsequently, YOLOv3 was formulated by enhancing the feature extraction backbone network Darknet53, resulting in an enhanced processing speed [18]. Bochkovskiy et al. [14] proposed YOLOv4 with the aim of fine-tuning the equilibrium between detection precision and processing speed. This iteration stood out as an exceptionally advanced detector, exhibiting swifter operation and heightened accuracy in comparison to existing alternatives. As the most recent addition to the YOLO lineage, YOLOv7 is architected around a trainable Bag of Freebies, thereby empowering real-time detectors to notably enhance accuracy without inflating inference expenses. Additionally, it substantially enhances detection velocity by curtailing parameter quantities and computational demands, achieved through the utilisation of “extend” and “compound scaling” strategies [19]. Emerging as the latest benchmark, YOLOv7 outperforms all existing object detectors in terms of both speed and accuracy [20]. In the context of identifying strawberry ripeness, the initial research conducted by Habaragamuwa et al. [21] introduced a Deep Convolutional Neural Network (DCNN). This network was designed to distinguish between two categories of strawberries—mature and immature—utilising images from greenhouses. The resulting deep-learning model attained an average precision of 88.03% for mature strawberries and 77.21% for immature strawberries. More recently, Y. Wang et al. [22] proposed a multi-stage approach for detecting strawberry fruits using YOLOv3, resulting in a mean average precision (mAP) of 86.58% and an F1 Score of 81.59%. Despite their capacity to discern strawberry ripeness, these prior investigations have yet to be evaluated in real-time conditions owing to their sluggish detection speeds. Consequently, the task of identifying the ripeness of strawberry fruits in practical field settings remains demanding, as evidenced by the modest detection accuracies (i.e., mAP < 90%).

Significant opportunities lie in the integration of emerging digital technologies within the agri-food industry. Augmented reality (AR) technology, in particular, facilitates the overlay of computer-generated virtual information onto the physical world [23]. The utilisation of AR technology has the potential to bring about a revolutionary change in agricultural applications by enriching the physical world with immersive virtual information, thus overcoming human limitations. In recent times, there has been an increase in the volume of literature focusing on the integration of AR within precision farming practices. For instance, Goka et al. [24] proposed a harvest support system aimed at aiding tomato harvesting, utilising Microsoft HoloLens for visualising the sugar content and acidity levels of individual tomatoes. Additionally, augmented reality techniques have been employed to facilitate the identification of weeds [25], plants [26], and pests [27], where users could be guided to the location that needs intervention. AR also enables the representation of Internet of Things (IoT) information, which can be overlaid onto an actual crop in real time. This approach enables farmers to engage with IoT data seamlessly within the real-world setting. As a result, it significantly enhances monitoring duties and minimises expenses related to planting operations. Consequently, it is clear that AR technology plays a pivotal role in the advancement of agriculture by enhancing the effectiveness and output in the administration of farming tasks.

Finally, the introduction of autonomous harvesting robots is costly and, therefore not a viable option for many small business-sized growers. At present, most farmers harvest strawberries manually based on human observation to decide the level of ripeness, leading to an uncertain harvest quality. Hence, in this work, we aim to develop an AR head-mounted display system that captures images of strawberries and displays the predicted ripeness label in real-time by leveraging cutting-edge deep learning technology (i.e., YOLOv7) for the rapid detection of strawberry ripeness in the greenhouse. In summary, the key objectives of this work are (a) developing an object detector for the ripeness identification of strawberries, (b) testing the model performance using a different variety of strawberries for in-field validation, and (c) designing an AR application framework for the real-time detection of the ripeness levels of strawberry fruit in the greenhouse. The primary novelty of this work lies in the integration of AR technology with object detection for the real-time identification of strawberry ripeness. While object detection has been used extensively for various applications, its combination with AR for fruit ripeness assessment is a pioneering concept. This integration offers a visually intuitive and contextually informative platform, bridging the gap between digital predictions and real-world scenarios.

## 2. Materials and Methods

### 2.1. Strawberry Images

The strawberry images used in this study were kindly provided by the StrawDI team [28] (see https://strawdi.github.io/, accessed on 8 July 2022). The dataset encompasses 8000 strawberry images sourced from 20 plantations spanning around 150 hectares in the province of Huelva, Spain. Notably, these photographs were taken amidst their organic growth circumstances, within a harvest period that extended from mid-December 2018 to early May 2019. To acquire the strawberry images, a Samsung Galaxy S7 Edge smartphone was linked to an extendable arm. This image-gathering process occurred across varying degrees of natural daylight brightness to introduce diversity to the dataset.

From the complete image pool, the StrawDI team chose a random subset of 3100 images. These selected images were then resized to dimensions of 1008 × 756. Following this, a data partitioning technique was applied, resulting in the creation of training (2800 images), validation (100 images), and test (200 images) sets. The provided dataset contained the ground truth for the instance segmentation of each strawberry fruit in terms of a mask. Annotations were subsequently established by creating a bounding box for each mask and manually assigning a label using MATLAB (release R2022a, The MathWorks, Inc., Natick, MA, USA). In this work, the ripeness levels are defined as “unripe”, “partially ripe”, and “ripe”, in which “unripe” refers to green or white strawberries, “partially ripe” represents the partial redness of the green strawberries, and “ripe” denotes uniformly red strawberries. Three representative images for each class are shown in Table 1. It is also noted that the unripe class had a considerably higher number of observations compared to the other two classes, presenting an imbalanced classification problem for modelling. Two authors participated in the data annotation; that is, one author implemented the image labelling process, the results of which were validated by the other author.

### 2.2. Object Detection Modelling

#### 2.2.1. Data Augmentation

**Figure 1:**
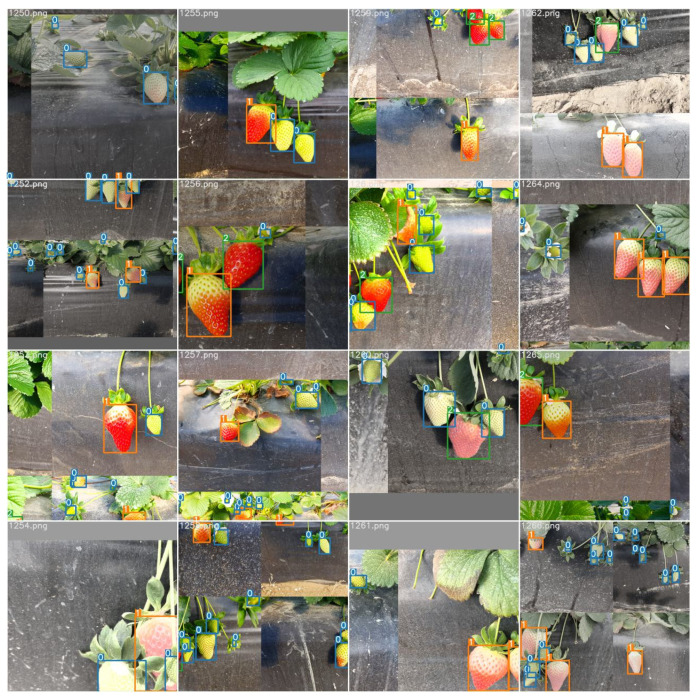
The augmented training images; 0, 1 and 2 represent the unripe, partially ripe and ripe strawberries, respectively.

Data augmentation can increase data variance for training, which has the potential to enhance model performance in terms of mitigating the model overfitting and increasing the generalizability of the developed model. In this work, the image augmentation methods included brightness change, image rotation, translation, scale, shear and perspective, horizontal and vertical mirroring, mosaic, mix-up, copy–paste and paste in. The parameters used for data augmentation are listed in Table 2. The variations in the complex lighting conditions contributed to disparities in the strawberry fruit images, leading to a potential interference with the detection outcomes. To counter this challenge, adjustments were made to the values of the three HSV channels (hue, saturation, and value) to represent distinct brightness levels. Simultaneously, techniques involving image transformation and mirroring (both horizontally and vertically) were implemented. For mosaic data augmentation, four images were randomly chosen and integrated into a single image following random scaling and cropping. This approach enhanced the richness of the training data within each batch size without escalating the batch size itself, thereby mitigating the GPU’s video memory consumption. The mix-up method employed linear interpolation to construct fresh training samples and corresponding labels, effectively expanding the dataset. Additionally, the training dataset’s scope was broadened through the copy–paste method, which generated supplementary training data by randomly duplicating a subset of instances from one image and embedding them onto other images. Examples of augmented images can be found in Figure 1.

#### 2.2.2. YOLO v7 Network Architecture

YOLOv7 is the latest in the family of YOLO models, and it is known for its fast detection speed and high precision, surpassing the currently available object detectors [19]. The YOLO framework comprises three primary components: the Backbone, Head, and Neck. The Backbone extracts crucial features from the input image and forwards them to the Head through the Neck. The Neck’s role is to accumulate feature maps derived from the Backbone and produce feature pyramids. The Head, positioned within the network’s structure, employs output layers to predict both the locations and categories of objects, delineating the bounding boxes around them. In comparison to its YOLO predecessors, YOLOv7 introduces several structural enhancements. To perpetually boost the network’s learning capability without disrupting the initial gradient pathway, YOLOv7 introduces the Extended Efficient Layer Aggregation Network (E-ELAN) as a computational block within the Backbone. This is achieved through the utilisation of the expand, shuffle, and merge cardinality techniques. YOLOv7 adopts a compound model scaling method to maintain synchronisation between the network depth and width for concatenation-based models. Furthermore, YOLOv7 incorporates several Bag of Freebies techniques aimed at elevating model performance without adding to the training cost [20]. In the proposed reparametrized convolution approach, the convolution layer within the E-ELAN computational block is replaced by a RepConv that lacks an identity connection. This substitution enhances the gradient diversity across various feature maps. YOLOv7 introduces a multi-headed framework, where the primary output-producing head is termed the lead head, while the auxiliary head contributes to training support. Collectively, these advancements have resulted in substantial enhancements in capability and cost reduction when contrasted with previous iterations. YOLOv7-tiny emerges as a streamlined variant of YOLOv7.

#### 2.2.3. Transfer Learning, Fine-Tuning, and Model Training

**Figure 2:**
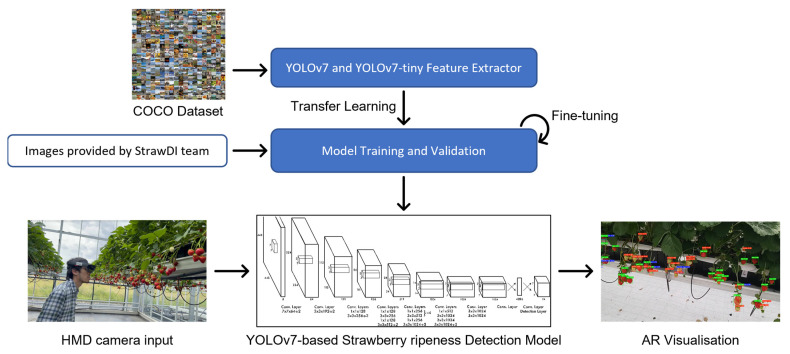
The schematical diagram for training and executing the YOLO models.

Utilising the PyTorch deep learning framework, the training process took place on a desktop computer equipped with an AMD Ryzen 9 5950X CPU, along with an NVIDIA GeForce RTX 3090 boasting 35.58TFLOPS, 24 GB of video memory, and 64 GB of RAM. The modelling development was conducted in Ubuntu on Windows Subsystem for Linux (WSL) running Python v3.10.8, PyTorch v1.13.0, and CUDA v11.7.99. Training a DL model from scratch is typically infeasible for certain applications because it requires enormous amounts of labelled data. In response to this challenge, transfer learning presents itself as a viable strategy to mitigate the necessity for an extensive volume of training data and minimise the training duration by leveraging a pre-trained model as the initial foundation [29]. In this work, we leveraged transfer learning by using pre-trained weights from the Microsoft COCO dataset [30] to enhance our model’s performance at detecting strawberries’ ripeness. We also applied fine-tuning to optimise the pre-trained model further (see Figure 2), ensuring efficient resource allocation and preventing memory depletion during training [31]. Of greater significance, hyper-parameters, encompassing elements such as batch size, subdivisions, learning rate, momentum, decay, and iterations, were meticulously customised.

When functioning at a singular scale, YOLO encounters challenges in detecting small objects within images of high resolution. To overcome this limitation, the multi-scale training of YOLO has been proposed, which involves training the algorithm on images of various scales to improve its ability to detect small objects. In this work, we investigated the effectiveness of multi-scale training of YOLOv7 and compared its performance with the standard YOLO algorithm on benchmark datasets. Meanwhile, the tiny YOLO has been introduced to increase detection speed by using fewer layers and smaller filters to reduce the number of computations needed for object detection. In this work, we explored the balance between detection speed and accuracy within the context of the tiny YOLO. 

Fine-tuning is a specific aspect of transfer learning. It refers to the process of adjusting the parameters of a pre-trained model on a new dataset or task. In this work, the optimal hyper-parameters of a YOLO model were determined via a systematic exploration of hyper-parameter combinations within specified ranges. The procedure started with the identification of the hyper-parameters that significantly affect the YOLO model’s performance. Subsequently, ranges or discrete values for each hyper-parameter that needed to be searched over were defined. A number of random combinations to explore from the hyper-parameter space were determined, and the corresponding model was trained based on each hyper-parameter combination. After training and evaluating the model for all combinations, the set of hyper-parameters that resulted in the best performance was identified. The training epoch was set to 100, while Table 3 demonstrates the main hyper-parameters for each model configuration. Over the training process, Weights & Biases (https://wandb.ai) was used to record and visualise the results, as well as to assess the performance of the model.

#### 2.2.4. Evaluation Metrics

The YOLOv7 neural network was developed using the training set, while evaluation metrics were derived from the validation set, employing specific model weights. Subsequently, model weights exhibiting optimal performance were chosen as the initial model. The test set was employed to gauge the model’s effectiveness when applied to new data, thereby indicating its generalisation capability. Within this study, the objective assessment of the model’s performance relied on precision (P), recall (R), mean average precision (mAP), and F1 score. Detection speed was evaluated based on the average frame time. Precision, being a widely adopted evaluation measure, is computed as follows:

(1) $$P=\frac{TP}{TP+FP}$$

where TP (True Positive) signifies the count of positive samples correctly identified, and FP (False Positive) denotes the count of samples inaccurately classified as positive.

Recall (alternatively referred to as sensitivity) gauges the capacity of a model to accurately predict positive instances out of all the positive cases within the dataset. Its calculation follows the equation

(2) $$R=\frac{TP}{TP+FN}$$

where FN (false negative) designates a situation where the model makes an incorrect prediction in the negative class.

Meanwhile, AP serves as a widely adopted metric for the assessment of object detection models, calculated as the weighted average of precisions across various thresholds. The overall mAP is determined by averaging AP values across all classes.

(3) $$AP=\int_{0}^{1}P\left(r\right)dr$$

(4) $$mAP=\frac{1}{n}\sum_{i=1}^{n}{AP}_{i}$$

The F1 score evaluates the harmonic mean of precision and recall, enabling a balanced consideration of metrics when precision or recall values are notably small. Computation of the F1 score is determined using the following equation:

(5) $$F1=\frac{2\times P\times R}{P+R}$$

### 2.3. Proposed AR Application Framework

#### 2.3.1. AR Headset

As the successor to the Microsoft HoloLens (1st gen), HoloLens 2 is integrated with a range of different sensors and functionalities such as the RGB camera, the depth camera, head tracking, hand tracking, and eye tracking. This untethered optical see-through (OST) head-mounted display (HMD) provides a more comfortable and immersive mixed-reality experience, enabling the wearer to move freely while collecting and processing imaging data, and ultimately presenting virtual data in the real world.

#### 2.3.2. DL Model Executing

This work employed the Unity Barracuda inference engine for YOLOv7 execution. Barracuda stands as a Unity package capable of executing Neural Network (NN) models directly on the device. Leveraging the device’s accessible GPU and CPU resources, it undertakes the computation of network operations defined in the ONNX format. This entails assessing and contrasting the latency through the computation and comparison of input image resolutions. This work conducted a comparative analysis of the average detection times for various deep learning models, subsequently evaluating the real-time efficacy of these models.

#### 2.3.3. AR Implementation

**Figure 3:**
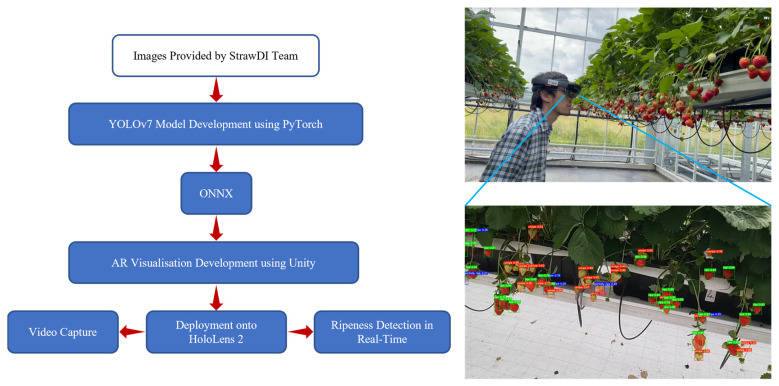
The schematic diagram for the proposed AR application framework.

Figure 3 presents the proposed integrated framework integrating deep learning and augmented reality technology. To begin with, the YOLOv7 model was developed using an online database provided by the StrawDI team. The pre-trained model was then directly exported in the Open Neural Network Exchange (ONNX) format, which enables developers to share DL models between different platforms. The pre-trained model was subsequently executed in Unity. The AR application was developed within Unity on 30 March 2020, utilising the most recent Windows 10 SDK along with certain components and features from Mixed Reality Toolkit (MRTK) 2. During the project’s compilation in Unity, the designated platform was UWP, with the intended device being HoloLens, employing ARM64 architecture. The application was tested in the strawberry greenhouse of Ashtown Food Research Centre, Teagasc, Ireland. The first proposed implementation method involved using both Hololens and HoloLens 2 to capture the footage to simulate the AR visualisation. The second approach aimed to realise the ripeness detection in real time. The HMDs captured RGB images from the user’s perspective and forwarded them to a pre-trained model for the acquisition of detection outcomes. The predicted outputs included bounding boxes and the predicted classes were overlayed onto the real world, allowing DL to be integrable in the augmented reality application.

## 3. Results and Discussion

### 3.1. Training Process and Fine-Tuning

**Figure 4:**
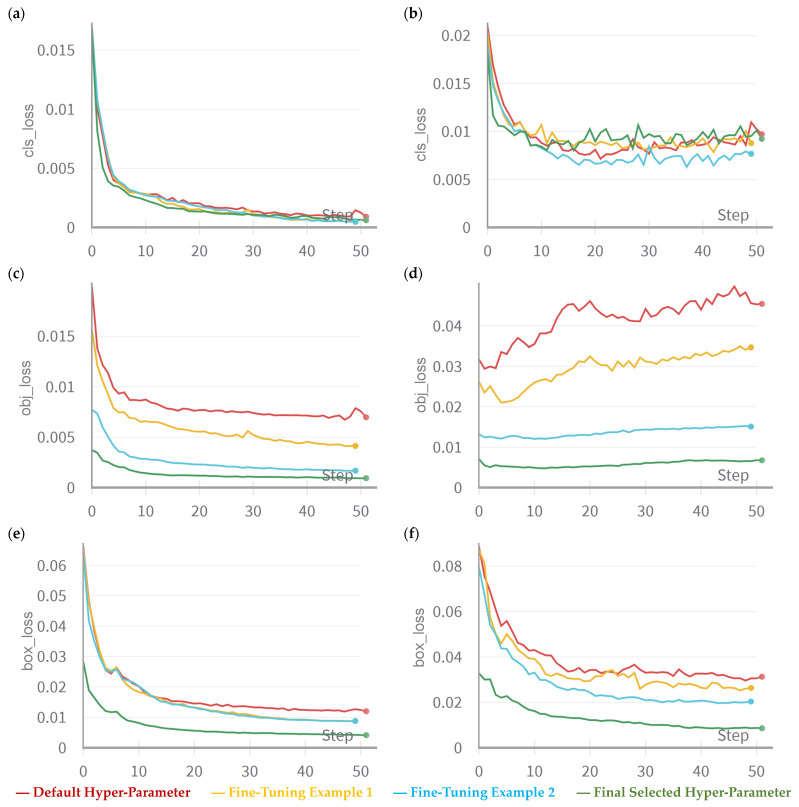
Example of the fine-tuning practice and iterations comparing the loss curves of the default hyper-parameter, two examples after fine-tuning, and the final selected hyper-parameter. For the training set, the classification loss, objectness loss, and box loss are presented in (**a**,**c**,**e**), respectively. For the validation set, the classification loss, objectness loss, and box loss are presented in (**b**,**d**,**f**), respectively.

Fine-tuning involves adjusting the existing model’s parameters to better suit the specific task without modifying the underlying architecture. Figure 4 showcases an example of the fine-tuning practice in this work. The classification loss evaluates the accuracy of the model’s prediction regarding the correct class of a detected object. Objectness pertains to the likelihood of an object’s presence within a designated region of interest. When objectiveness is high, it indicates the probability of an object’s presence within an image window. Consequently, objectness loss is commonly referred to as confidence loss. On the other hand, the box loss, also termed as localisation loss, gauges the disparities between the projected bounding box and the actual ground truth. As a result, it quantifies the model’s aptitude in pinpointing an object’s centre and accurately encompassing the object with the predicted bounding box. Using original parameters, the validation objectness loss keeps increasing during the training of a YOLOv7 model, which implies that the model is having difficulty distinguishing between the objects and background. The objectness loss weight is used to control how much emphasis the model places on correctly predicting whether an object is present in a given bounding box. Therefore, increasing the objectness loss weight could help the model focus more on object detection. As can be seen in Figure 4, the process of fine-tuning led to a desirable validation objectness loss trend.

**Figure 5:**
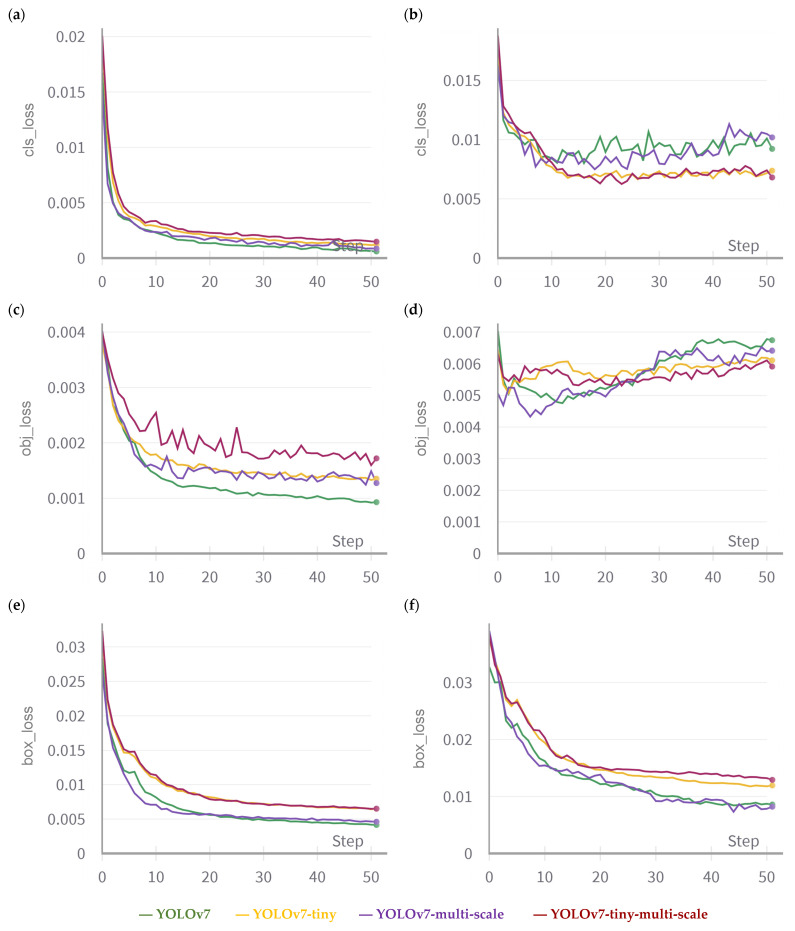
For the training set, the classification loss, objectness loss, and box loss are presented in (**a**,**c**,**e**), respectively. For the validation set, the classification loss, objectness loss, and box loss are presented in (**b**,**d**,**f**), respectively. Multi-scale refers to the model trained on images of various scales. Tiny means a model using fewer layers and smaller filters to reduce computation time.

**Figure 6:**
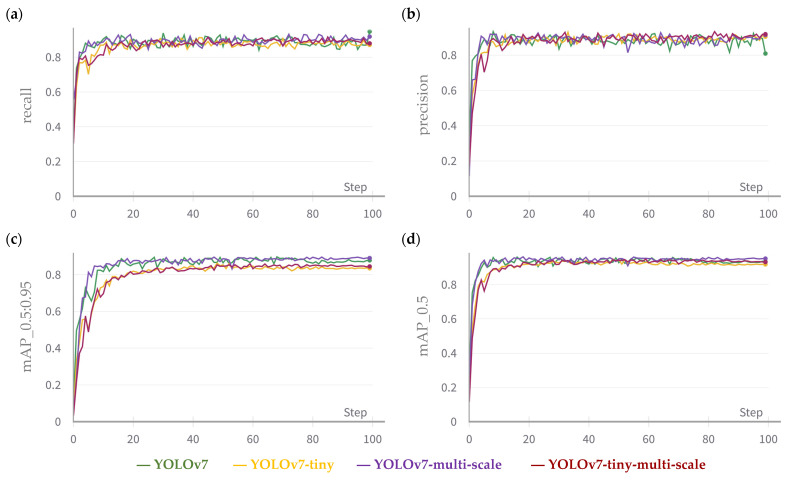
The evolution of recall (**a**), precision (**b**), mAP at 0.5:0.95 (**c**), and mAP at 0.5 (**d**) during the training process. Note: multi-scale refers to the model trained on images of various scales. Tiny means a model using fewer layers and smaller filters to reduce computation time.

After fine-tuning, the loss graphs can be visualized in Figure 5. During training, the training losses gradually decrease as the model gets better at correctly detecting objects in the training set. On the other hand, the validation losses can tell how well the model is generalising to new, unseen data. As evident, the models exhibited minimal loss during validation when tested against the image dataset. It is also noted that validation objectness loss initially decreases and then increases during training. This pattern can be elucidated by the observation that the model initially focuses on learning to detect objects that are comparably straightforward to recognise, such as large objects or objects with distinctive features. As the model continues to learn, it becomes more sensitive to smaller or more difficult objects, which can lead to an increase in objectness loss. Another possible explanation for the increase in the objectness loss is overfitting, which can be diagnosed by a model’s performance on the test set consisting of unseen images during training. Simultaneously, Figure 6 further demonstrates the model’s gradual improvement in precision and recall as training on the dataset continued. In contrast, when analysing the mAP at 0.5:0.95—which assesses the mean average precision across the intersection over union (IoU) thresholds spanning from 0.5 to 0.95—it becomes evident that the standard YOLOv7 and YOLOv7-multi-scale consistently exhibited better performances than the two compact models throughout the entire training duration. This observation suggests the superior prowess of the standard model in comparison to its lightweight counterparts. Furthermore, the mAP of 0.5, which focuses on the mean average precision metric solely at an IoU threshold of 0.5, conveys a similar message, albeit with a somewhat subdued differentiation between standard and tiny models. Together, we can see that standard YOLOv7 demonstrates a faster convergence speed and better convergence results compared to the tiny versions.

### 3.2. Overall Model Performance

Table 4 summarises the performance of the models developed in this work. According to the results, the YOLOv7-multi-scale had the highest mAP of 0.89 and F1 score of 0.92 for detecting the ripeness levels of strawberries. It is followed by the standard YOLOv7 model with an mAP of 0.88. Overall, YOLOv7 models have a higher mAP and F1 score than the tiny versions, in agreement with the evolution of the mAP across training iterations shown in Figure 6. The enhanced performance of standard models might be attributed to their larger architecture and increased computational complexity. We also notice that multi-scale training enables better model performance. By training the model with images of different resolutions, multi-scale training allows the model to better detect objects at varying sizes and scales, therefore increasing its ability to detect small and heavily occluded objects more accurately.

**Figure 7:**
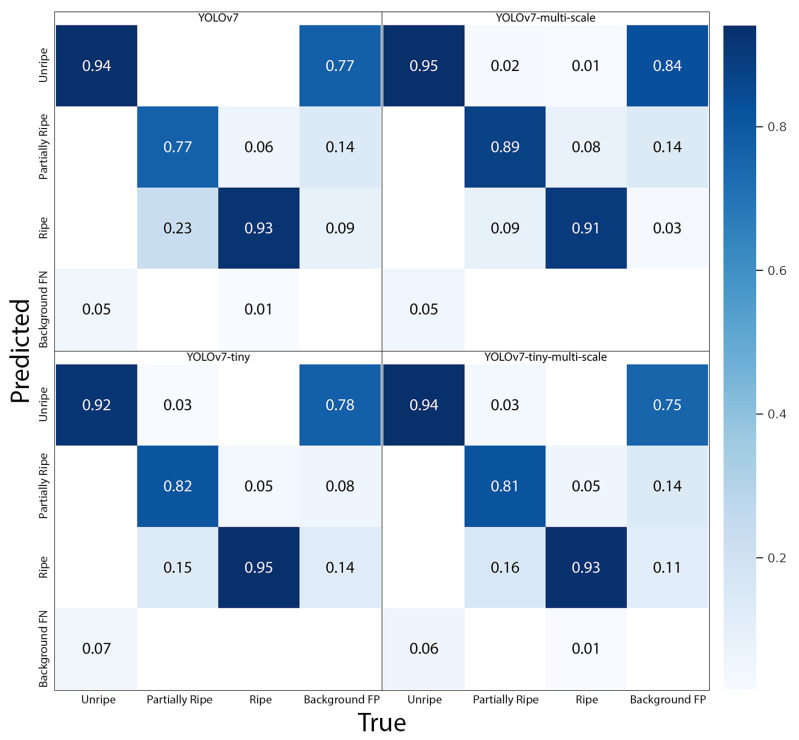
Normalised confusion matrices of the developed four models. Note: multi-scale refers to the model trained on images of various scales. Tiny means a model using fewer layers and smaller filters to reduce computation time.

Figure 7 illustrates the normalised confusion matrices of the four established models. A quick examination of the matrices reveals that the class of “partially ripe” strawberries presented the greatest challenge for detection, as a relatively high number were incorrectly classified as “ripe” strawberries. Additionally, it is apparent that the “unripe” class had the highest rate of background false positives, indicating a greater similarity between the background and unripe strawberries. Among the models, the YOLOv7-multi-scale excelled in identifying “unripe” and “partially ripe” strawberries, achieving accuracies of 0.95 and 0.89, respectively. However, this model also demonstrated the highest background false positive rate of 0.84, indicating a greater likelihood of incorrectly identifying part of the background as an unripe strawberry. Conversely, the YOLOv7-tiny-multi-scale was the most accurate at detecting “ripe” strawberries, with an accuracy of 0.95.

**Figure 8:**
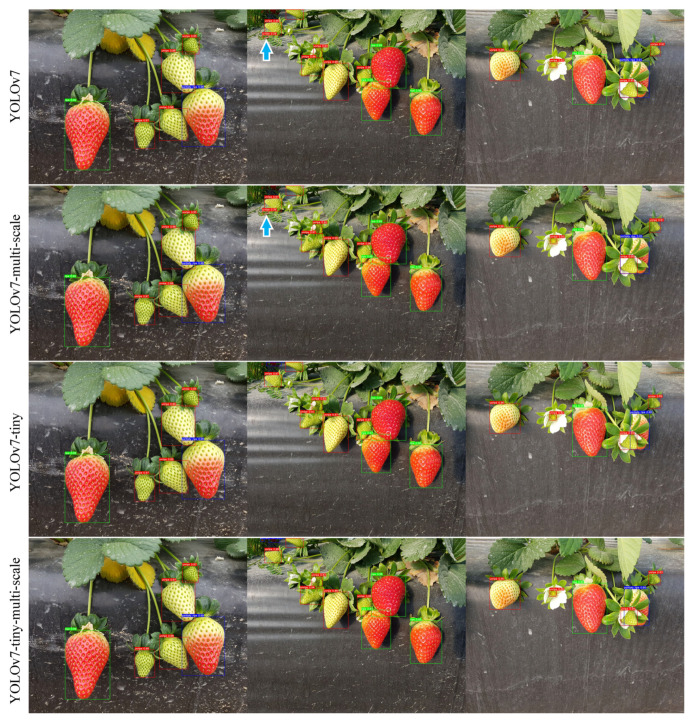
Examples of ripeness detection from randomly selected test images. Bounding boxes in red, blue, and green indicate “unripe”, “partially ripe”, and “ripe” strawberries, respectively. A blue arrow suggests the case when the model mistakenly detects a leaf as an unripe strawberry. Note: multi-scale refers to the model trained on images of various scales. Tiny means a model using fewer layers and smaller filters to reduce computation time.

Figure 8 shows the ripeness detection results from three randomly selected test images. The various developmental stages of strawberry fruits are represented by bounding boxes of distinct colours, with red indicating “unripe”, blue indicating “partially ripe”, and green indicating “ripe” strawberries. Overall, all models demonstrated a good performance, with the majority of strawberries being correctly identified. However, it is worth noting that the YOLOv7 and YOLOv7-multi-scale models mistakenly detected a leaf as an unripe strawberry (as is indicated by blue arrows in the figure), yet this was not observed in the tiny models.

### 3.3. Comparison with State-of-the-Art

We compared our results with previous studies that aimed to detect strawberries using object detection methods (see Table 5). However, a direct comparison was not possible due to differences in sample sizes and variations in the methods used in those studies. It is worth noting that only two studies [21,22] have specifically directed their attention towards employing object detection for the classification of strawberry ripeness levels, while the remaining studies have centred on detecting the fruits themselves. Our work achieved the highest mAP and F1 score using the advanced YOLOv7 model, outperforming the other methods employed in the previous studies. These observations indicate that YOLOv7 attains state-of-the-art outcomes in strawberry detection. 

### 3.4. AR Implementation

#### 3.4.1. AR Simulation

**Figure 9:**
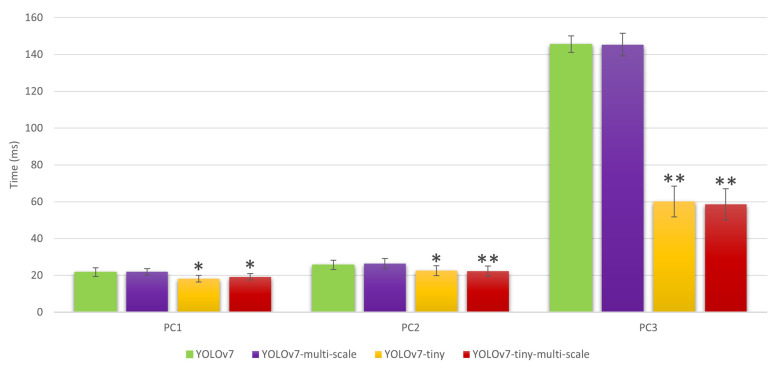
Detection speed(s) of individual models running on various computers (i.e., PC1, PC2, and PC3). * significant different from YOLOv7 model at *p* < 0.01, ** significant different from YOLOv7 model at *p* < 0.001.

A total of four video clips were captured in the strawberry greenhouse using both HoloLens (1216 × 684 resolution) and HoloLens 2 (1280 × 720 resolution). For implementation, each video clip was divided into individual frames. Then, each frame was fed into the YOLO models for object detection, which generates bounding boxes around the strawberry it detects in each frame. Once all frames were processed, the resulting video could be reconstructed by stitching the frames back together. A GPU was used for such processing. The experiment involved comparing the performance of three computers (specifications can be found in Table 6) in terms of object detection time, as is illustrated in Figure 9. A paired *t*-test was used to determine if the detection speed of the modified models was significantly different from that of the standard YOLOv7. It was found that the use of multi-scale training did not result in a significantly longer detection time. The findings also revealed that the tiny models had a significantly reduced detection time (*p* < 0.01). When exporting the models in the ONNX format, it was observed that the tiny models had a file size that was over 80% smaller than that of the standard YOLOv7 models.

**Figure 10:**
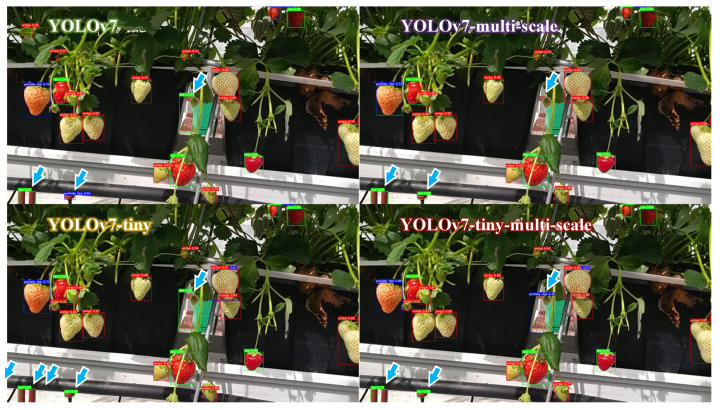
One scene of ripeness detection from one video clip captured in the Teagasc strawberry greenhouse. Bounding boxes in red, blue, and green indicate “unripe”, “partially ripe”, and “ripe” strawberries, respectively. A blue arrow suggests the misclassification scenario. Note: multi-scale refers to the model trained on images of various scales. Tiny means a model using fewer layers and smaller filters to reduce computation time.

The object detection performance of one video clip using the YOLOv7-tiny-multi-scale model is uploaded as the Supplementary Material. Within the video clips, occurrences of partial occlusion involving branches and leaves are frequent, along with instances of strawberry overlap. These factors are likely to exert an impact on the accuracy of strawberry detection. It is worth noting that the strawberry variety tested in Ireland differed from the training samples collected in Spain. Additionally, the background settings were vastly dissimilar, with the Irish samples being captured in a greenhouse and the Spanish samples in an open field. Furthermore, different image capture systems were used. Despite these variations, our models performed commendably by accurately detecting the majority of strawberries. Concurrently, the constructed models exhibited the capability to identify a substantial portion of strawberries that were occluded or overlapping, underscoring the tangible importance of the model introduced in this study. To compare the different models developed in this study, one scene was chosen, and their object detection performance is displayed in Figure 10. It indicates that some reddish objects in the scene (as indicated by blue arrows in the figure) were erroneously identified as ripe/partially ripe strawberries. This can be attributed to the absence of such obstructive backgrounds in the training samples, causing the model to be unable to acquire this knowledge. Notably, the YOLOv7-multi-scale model displayed a superior performance to the other models, with slightly fewer mistakes of this nature. Overall, the tiny models achieved outstanding performance across accuracy, detection speed, and memory utilisation.

#### 3.4.2. AR Real-Time Application

Despite the development of a resilient, efficient, and lightweight object detection model tailored for edge computing platforms like the HoloLens 2, intended to visualise strawberry detection and superimpose bounding boxes onto actual strawberries, several hurdles remain. Among these challenges, a key concern is achieving real-time data processing to guarantee a seamless and fluid overlay of bounding boxes onto the real strawberries. This involves capturing images from the camera, running object detection algorithms on these images, and overlaying the bounding boxes within a fraction of a second.

The Unity game engine was used as the core platform to develop the AR application for HoloLens 2 due to the widely supported and well-integrated Mixed Reality Toolkit 2 (MRTK2) developed by Microsoft. The Barracuda package stands as a lightweight, cross-platform neural network inference library designed for Unity, capable of executing neural networks on both the CPU and GPU. However, at the time of testing, Barracuda did not support inferencing on HoloLens 2’s GPU. Therefore, inferencing could only be performed on the CPU, resulting in a suboptimal performance with an inference time of over 1.5 s per frame even when using the tiny models, making it impossible to keep up with the movement of the strawberries in real-time. This challenge can only be resolved in the future when a neural network inference library supports inferencing the HoloLens 2 GPU.

Due to the nature of OST design, the RGB camera of the HoloLens 2 is offset above the viewpoint of the user. However, the output from the YOLOv7 only provides identification data on the 2D image input, making the identification labels offset by various degrees. Attempts were made by incorporating the depth information from the HoloLens 2, along with the CameraToWorld matrix and Projection matrix, to correct the offset.

Another challenge is related to the lighting conditions. When testing in bright sunlight, it was extremely difficult to see the bounding boxes overlaid in the real-world environment. We tried using contrasting colours such as yellow, but due to the complex background colours and challenging light conditions, the visualisation of the bounding box was not desirable. To address this challenge, the AR glasses would need to have a high-brightness display that provides enough contrast between the bounding box and the real-world environment. Furthermore, an adaptable brightness functionality could be integrated to automatically modify the display brightness in response to the prevailing ambient light conditions.

## 4. Discussion

In this study, we focused on the development of an innovative AR head-mounted display system that employs cutting-edge deep learning technology, specifically YOLOv7, to realise real-time strawberry ripeness detection within greenhouse environments. Our research distinguishes itself from existing methods in several key aspects. Traditional strawberry ripeness assessment methods often rely on manual observation and subjective judgment, leading to inconsistencies and delays in decision making. In contrast, our AR head-mounted display system provides an automated, real-time solution that mitigates human error and offers instantaneous insights into fruit ripeness. While various image-based fruit assessment techniques exist, the integration of AR technology and deep learning object detection for agricultural purposes is a unique departure from conventional practices. Furthermore, our approach goes beyond the confines of controlled laboratory conditions. The validation of our model using various strawberry varieties in real-world greenhouse environments demonstrates its robustness and adaptability. 

The proposed strawberry ripeness detection system exhibits certain limitations that warrant consideration. First of all, lighting conditions can significantly impact the colour representation in the images, which is a critical parameter for our RGB-based ripeness classification. To account for variations in lighting conditions and to enhance the model’s ability to generalise across diverse scenarios, we have implemented a data augmentation strategy that introduces adjustments to the values of the three HSV channels. Meanwhile, strawberries, often nestled amidst green foliage, can indeed pose challenges for accurate image-based classification. Leaves may occlude parts of the fruit, altering the colour appearance captured by the camera. As is seen in Figure 8, the YOLOv7 and YOLOv7-multi-scale models mistakenly detected a leaf as an unripe strawberry. Future work involves continually refining the object detection component of our system, leveraging YOLOv7’s robustness to handle occlusions and varying object sizes. Employing a random grid search for fine-tuning is a limitation in comparison to contemporary state-of-the-art practices. Random grid searches lack the assurance of discovering the globally optimal hyper-parameters. Further techniques, such as Bayesian optimisation, can be used for more advanced and targeted hyper-parameter tuning. Meanwhile, utilising Python’s HyperOpt library can simplify hyper-parameter tuning with sophisticated algorithms such as Tree-structured Parzen Estimators; therefore, it requires less manual intervention compared to a random grid search.

Other limitations include the dependence on specific hardware for AR applications, which might pose challenges in terms of availability and compatibility. This dependency could limit the widespread adoption of the proposed approach, especially in regions with limited access to specialized equipment. While the system has been initially validated in greenhouse settings, its performance in open-field conditions might differ due to variations in lighting and weather conditions, and potential interference from natural elements. Furthermore, the accuracy of the deep learning model is dependent on the quality of the annotated training data. Human errors during annotation could introduce inaccuracies that impact the model’s performance. In addition, the effectiveness of the system depends on the user’s ability to operate and interpret the AR display. Adequate training and familiarisation are essential to ensure accurate and consistent ripeness assessments.

The focus of this work lies in delivering a practical and real-time solution to replace the subjective nature of an immediate visual assessment of strawberry ripeness, introducing a more objective and efficient approach. However, it is important to note that considering a broader spectrum of factors such as soluble solids, phenols, and Vitamin C content will undoubtedly contribute to achieving a holistic characterisation of ripeness. The inclusion of multiple characteristics has the potential to facilitate the development of a comprehensive grading or sorting system, enabling farmers to make informed decisions based on harvested strawberry quality. Therefore, future research endeavours should be performed to refine this work by integrating these essential aspects and further enhancing the accuracy and applicability of this strawberry ripeness classification system.

One of the most immediate practical implications is the acceleration of the decision-making processes in strawberry cultivation. The ability to swiftly and accurately identify ripe strawberries reduces the time required for manual assessment. This is especially valuable in large-scale greenhouse operations where timely harvesting decisions can impact yield quality and minimise waste. More importantly, the implications of our work extend beyond immediate ripeness identification. The real-time overlay of predicted ripeness labels onto physical strawberries provides growers with targeted information. This can guide interventions such as selective harvesting or customised treatment strategies based on the specific needs of individual plants or areas. The successful integration of AR and deep learning opens avenues for broader applications in agricultural practices, including automated sorting and grading systems. 

## 5. Conclusions

In this research, we have introduced a real-time and precise detection approach employing the YOLOv7 target detection network to identify the various ripeness stages of strawberries. The results demonstrate that transfer learning and fine-tuning with YOLOv7 models can efficiently detect ripeness levels, and that multi-scale training leads to better detection performance. In addition, using tiny versions of the models can reduce memory storage and improve detection time, albeit with a slightly deteriorated detection performance. The proposed model performs robustly when applied to an independent test field. The study also evaluates the potential of AR systems to visualise bounding boxes on real strawberries in real time. However, technical challenges were identified for practical use by farmers during harvesting due to latency and difficulties in visualising bounding boxes under challenging lighting conditions. Future work aims to optimise the AR framework by conducting data processing analysis on a cloud server and using 5G network communication.

## Figures and Tables

**Table 1:** Summary of the dataset used for object detection modelling.

Labels	Representative Images	Number of Strawberry Fruits		
		Training	Validation	Test
Unripe	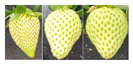	11,674	405	816
Partially ripe	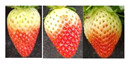	2729	81	177
Ripe	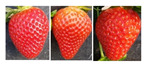	1831	86	139

**Table 2:** Data augmentation parameters of YOLOv7.

Parameter	Descriptions	YOLOv7	YOLOv7-Tiny
HSV_H	HSV-Hue augmentation (fraction)	0.015	0.015
HSV_S	HSV-Saturation augmentation (fraction)	0.7	0.7
HSV_V	HSV-Value augmentation (fraction)	0.4	0.4
Degrees	Image rotation (+/- deg)	0.0	0.0
Translate	Image translation (+/- fraction)	0.2	0.1
Scale	Image scale (+/- gain)	0.5	0.5
Shear	Image shear (+/- deg)	0.0	0.0
Perspective	Image perspective (+/- fraction)	0.0	0.0
Flipud	Image flip up–down (probability)	0.0	0.0
Fliplr	Image flip left–right (probability)	0.5	0.5
Mosaic	Mosaic (probability)	1.0	1.0
Mixup	Mix-up (probability)	0.0	0.05
Copy_paste	Copy–paste (probability)	0.0	
Paste_in	Copy–paste (probability)	0.0	

**Table 3:** Hyper-parameters of the fine-tuned models.

Hyper-Parameter	YOLOv7	YOLOv7-Multi-Scale	YOLOv7-Tiny	YOLOv7-Tiny-Multi-Scale
Batch size	32	16	32	32
Initial learning rate	0.01	0.01	0.01	0.01
Momentum	0.937	0.937	0.937	0.937
Weight decay	0.0005	0.0005	0.0005	0.0005
Box loss gain	0.02	0.02	0.02	0.02
Classification loss gain	0.3	0.3	0.3	0.3
Objectness loss gain	0.1	0.1	0.1	0.1
IoU training threshold	0.2	0.2	0.2	0.2
Anchor-multiple threshold	4.0	4.0	4.0	4.0

**Table 4:** Comparison of the overall detection performance for mAP and F1 score.

Metrics	YOLOv7	YOLOv7-Multi-Scale	YOLOv7-Tiny	YOLOv7-Tiny-Multi-Scale
F1 score	0.87	0.92	0.89	0.90
mAP	0.88	0.89	0.84	0.85

**Table 5:** Comparison with existing object detection approach for strawberries.

Method	Application	Input Size	mAP	F1 Score
Mask R-CNN [28]	Strawberry instance segmentation	768 × 1005	0.45	-
DSE-YOLO [22]	Multi-stage ripeness of strawberry detection	608 × 608	0.87	0.82
YOLOv3 [32]	Strawberry detection	104 × 104	0.83	0.81
YOLOv3-tiny [32]	Strawberry detection	104 × 104	0.75	0.71
YOLOv4 [32]	Strawberry detection	104 × 104	0.84	0.82
YOLOV4-tiny [32]	Strawberry detection	104 × 104	0.83	0.79
CNN [33]	Strawberry detection	360 × 640	0.88	-
DCNN [21]	Mature and immature strawberry detection	-	0.83	-
Our best model	Multi-stage ripeness of strawberry detection	Multi-scale training	0.89	0.92

Note: DSE: Detail-Semantics Enhancement; R-CNN: Mask Region Convolutional Neural Network.

**Table 6:** Computer specifications.

Computers	Manufacture Year	CPU	GPU	RAM
PC1	2022	AMD Ryzen 9 5950X	NVIDIA GeForce RTX 3090	64 GB
PC2	2021	Intel Core i7 11800H	NVIDIA GeForce RTX 3070	64 GB
PC3	2015	Intel Core i7 5500U	NVIDIA GeForce GTX 960M	8 GB

## Data Availability

The data presented in this study are available on request from the corresponding author.

## Supplementary Materials

The following supporting information can be downloaded at: https://www.mdpi.com/article/10.3390/s23177639/s1. The object detection performance of one video clip using YOLOv7-tiny-multi-scale.
